# Microsurgical treatment of a posterior fossa arteriovenous malformation initially mistaken for a dural arteriovenous fistula: avoidance of near disaster

**DOI:** 10.3171/2020.10.FOCVID2047

**Published:** 2021-01-01

**Authors:** Brian M. Howard, Daniel L. Barrow

**Affiliations:** 1 Department of Neurosurgery, Emory University School of Medicine; and; 2 Department of Radiology and Imaging Sciences, Division of Interventional Neuroradiology, Emory University School of Medicine, Atlanta, Georgia

**Keywords:** arteriovenous malformation, microsurgery, embolization, dural arteriovenous fistula

## Abstract

Many brain arteriovenous malformations (AVMs) derive dural blood supply, while 10%–15% of dural arteriovenous fistulas (dAVFs) have pial arterial input. To differentiate between the two is critical, as treatment of these entities is diametrically opposed. To treat dAVFs, the draining vein(s) is disconnected from feeding arteries, which portends hemorrhagic complications for AVMs. The authors present an operative video of a subtle cerebellar AVM initially treated as a dAVF by attempted embolization through dural vessels. The lesion was subsequently microsurgically extirpated. The authors show a comparison case of an AVM mistaken for a dAVF and transvenous embolization that resulted in a fatal hemorrhage.

The video can be found here: https://youtu.be/eDeiMrGoE0Q

## Transcript

**0:22 Presenting History.** The case is that of an otherwise healthy 18-year-old patient who presented to a referring facility with acute-onset worst headache of life associated with emesis and diplopia. Axial imaging demonstrated a right cerebellar hemispheric intraparenchymal hemorrhage. The patient was stabilized and taken for diagnostic angiography.

**0:36 Key Considerations.** Key considerations for this case include the differential diagnosis for cerebellar intraparenchymal hemorrhage with an emphasis on diagnostic accuracy to ensure that the appropriate therapeutic modality is employed.

**0:46 Pretransfer Imaging.** Right vertebral angiography demonstrates a subtle shunting lesion with a nidus in the superior aspect of the right cerebellar hemisphere at the petrosal surface [open arrow] with hemispheric cortical venous drainage to the superior petrosal vein [solid arrows]. The right external carotid artery injection elicited concern that the middle meningeal artery contributed to the arteriovenous malformation. Selective catheterization of the middle meningeal artery demonstrated arteriovenous shunting within the CP angle via the petrosal branches [arrowhead, nidus; arrows, draining vein]. An attempt was made to embolize the arteriovenous malformation via the middle meningeal artery using Onyx. Postembolization angiography of the right vertebral artery demonstrated persistence of the arteriovenous malformation. An attempt was made to selectively catheterize PICA to further embolize the lesion, but this was unsuccessful. Axial imaging following embolization demonstrates Onyx within the right middle meningeal artery from the foramen spinosum [green arrow] through the petrosal branches [yellow arrow] and into the cerebellar parenchyma [red arrow].

**1:59 History Upon Transfer, Rationale for Treatment.** The patient was referred to our facility for continued treatment of the AVM. After review of the case and informed consent, a right retrosigmoid craniotomy was pursued for microsurgical extirpation of the arteriovenous malformation.

**2:13 Right Retrosigmoid Craniotomy, AVM Resection.** Following a standard right retrosigmoid craniotomy, the confluence of the petrosal and tentorial surfaces of the cerebellum was exposed. After initial microdissection, the anterolateral marginal vein and the vein of the cerebellopontine fissure appear to be arterialized as they join to make the superior petrosal vein. The nidus is initially dissected from the superomedial surface near the tentorium inferiorly and laterally toward the cerebellopontine angle. Standard microsurgical technique is used and feeding arteries are coagulated and divided before they enter the nidus. The vein of the cerebellopontine fissure is the primary venous egress of the AVM, and care is taken to ensure that it remains intact until final nidal dissection. Once the AVM nidus has been circumferentially mobilized, the superior petrosal vein is coagulated and divided. The final pial and arachnoidal microdissection is completed sharply with microscissors to expose the fifth cranial nerve and seventh-eighth cranial nerve complex. The AVM is then removed.

**3:50 Intraoperative Angiogram.** Prior to closure, an intraoperative angiogram is completed to ensure that the entire AVM has been removed.

**4:01 Companion Case.** A similar case emphasizes the importance of the correct diagnosis in the treatment planning for intracranial arteriovenous shunting lesions. To differentiate pial arteriovenous malformations and pial AVMs with dural blood supply^1–3^ from dural arteriovenous fistulae is critically important, as premature occlusion of the draining vein of pial AVMs risks hemorrhagic complications.^4–6^ While differentiating between the two can be difficult, the primary determining characteristic is the location of the nidus, be it in a dural leaflet or the parenchyma. In this older case, the external carotid artery injection, which is not currently available, led the interventionist to believe this was a dural arteriovenous fistula and treated with transvenous coiling, which led to hemorrhagic complication and death.

## Acknowledgments

We thank Dr. Jacques Dion for providing the historical case presented for comparison.

## Author Contributions

Primary surgeon: Barrow. Editing and drafting the video and abstract: both authors. Critically revising the work: both authors. Reviewed submitted version of the work: both authors. Approved the final version of the work on behalf of all authors: Howard. Supervision: Barrow.
